# Early visual ERPs show stable body-sensitive patterns over a 4-week test period

**DOI:** 10.1371/journal.pone.0192583

**Published:** 2018-02-13

**Authors:** Katie Groves, Steffan Kennett, Helge Gillmeister

**Affiliations:** Department of Psychology, University of Essex, Colchester, United Kingdom; Radboud Universiteit, NETHERLANDS

## Abstract

Event-related potential (ERP) studies feature among the most cited papers in the field of body representation, with recent research highlighting the potential of ERPs as neuropsychiatric biomarkers. Despite this, investigation into how reliable early visual ERPs and body-sensitive effects are over time has been overlooked. This study therefore aimed to assess the stability of early body-sensitive effects and visual P1, N1 and VPP responses. Participants were asked to identify pictures of their own bodies, other bodies and houses during an EEG test session that was completed at the same time, once a week, for four consecutive weeks. Results showed that amplitude and latency of early visual components and their associated body-sensitive effects were stable over the 4-week period. Furthermore, correlational analyses revealed that VPP component amplitude might be more reliable than VPP latency and specific electrode sites might be more robust indicators of body-sensitive cortical activity than others. These findings suggest that visual P1, N1 and VPP responses, alongside body-sensitive N1/VPP effects, are robust indications of neuronal activity. We conclude that these components are eligible to be considered as electrophysiological biomarkers relevant to body representation.

## Introduction

In a pioneering study [1], a bilateral region in the lateral occipito-temporal cortex (extrastriate body area; EBA) was identified as a module for body processing as it was found to respond strongly and selectively to images of the human body and human body parts. Subsequent research has revealed that EBA activity is likely involved with processing body parts and body shape as well as possibly distinguishing identity and emotion [2, 3]. The fusiform body area (FBA), a second body-sensitive region found ventrally on the fusiform gyrus, was described a few years later [4]. It has been suggested that FBA and EBA contribute functionally distinct representations of the body to person perception [5] although this is somewhat debated [6–8]. Nonetheless, it is widely accepted that the visual perception of human bodies recruits highly specialised and selective neuronal networks in the occipital cortex [2].

Event related potential (ERP) studies have corroborated these findings, with reports of a functional difference in early electrophysiological responses over occipito-parietal and fronto-central electrodes when bodies are viewed in comparison to other stimuli [9]. This study was particularly interested in these early electrophysiological indicators of body representation. Temporal differences between responses to bodies and other stimuli have been reported as early as 100 ms after stimulus onset [10]. Most typically however, body-sensitive ERP responses are reported as an enhanced negative deflection peaking at around 150 ms– 190 ms after body viewing in comparison to viewing non-body stimuli [10–13]. Evidence from source localisation techniques [10, 14], direct intracranial recordings [12], magnetoencephalography [15] and transcranial magnetic stimulation (TMS) [16] has implicated EBA activity in the origins of this effect. The reported timing of the component has seen body-sensitive responses variably referred to as N1, N170 or N190 [3, 17]. We will refer to this component as a body-sensitive N1 throughout this paper.

In addition, reports have described a body-sensitive enhancement of the vertex positive potential (VPP), evident over fronto-central sites, when participants view bodies in comparison to non-body stimuli [16, 18, 19]. Studies on the selective processing of faces suggest that VPP responses are generated by the same neural sources as N1 responses and thus reflect the same process [20]. Although this has been questioned [21, 22], TMS delivered to EBA has been found to increase VPP amplitudes to bodies but not to faces, whereas TMS delivered to the occipital face area (OFA) resulted in the reverse pattern [16]. This therefore suggests that, similarly to the body-sensitive N1, body-sensitive VPP responses may also arise from EBA activity.

Investigations into body-sensitive ERP effects have typically focused on whether responses are modulated by emotion, and whether they provide an indication of the visual processing mechanisms employed during body perception (i.e., whether bodies are processed as a sum of their parts or whether they are processed on a feature-by-feature basis) [11, 13, 18, 19, 23]. The value of these findings is heavily reliant on the validity and reliability of the ERPs measured; in other words, the extent to which they can be considered trustworthy. A trustworthy measure must be both reliable (yields the same outcome when repeated) and valid (measures only what it claims to measure), with the validity of a scientific measurement dependent on its reliability [24].

Reliability is a serious concern for ERP research [25], important not only to establish whether reported effects are trustworthy, but also in order to ascertain whether ERPs and their associated effects are stable. It is therefore surprising that research into the reliability of visual ERPs is sparse. To the best of our knowledge, no study has investigated the test-retest reliability of body-sensitive ERP effects, whilst very few studies have investigated the test-retest reliability of early visual ERP components more generally [26–28]. This is a rather worrying oversight as ERP studies produce some of the most cited papers in the field of face and body representation. Not only this, but the electroencephalogram (EEG) is already a useful tool in the clinical, routine assessment of neurological conditions such as cerebrovascular disease, dementia and epilepsy [29]. In addition, at least two studies have reported encouraging results for the use of ERPs as ‘neuromarkers’ or ‘vital signs’ of cognitive (dys)function [30, 31]. Thus, with a move towards more objective neuropsychiatric evaluation techniques gathering momentum, ERPs have been proposed as a promising tool for the assessment of cognitive processes [32]. In particular, preliminary evidence suggests that early visual processing as indexed by P1 and N1 components, may provide a ‘bio-signature’ of an important phenotype in disorders of body image such as anorexia, bulimia and BDD [33]. Furthermore, it has been suggested that gender-sensitive body processing in N1 and VPP amplitudes may be potential ERP markers of body image disturbances in both bulimia and anorexia [34].

With reports stating that anorexia nervosa has the highest mortality rate of all psychiatric conditions [35, 36], there have been calls to implement more evidence-based treatments and early interventions [37]. The identification of objective, biological markers of ED symptoms is therefore imperative work. However, it will be fruitless if such measures cannot be applied because the reliability of early visual ERPs and their associated body-sensitive effects has not been established.

Thus, the primary aim of this study was to address the test-retest reliability of early visual ERPs, namely the P1, N1 and VPP, as well as the test-retest reliability of body-sensitive ERP effects. The reason for this was not only to inform the validity of research in the body processing field, but also to address whether these components have the potential to be trustworthy neural markers. With that in mind, participants were invited to complete the same image classification task, once a week for four consecutive weeks, (as stable visual responses have been found within this time frame, [38]) whilst we recorded their brain’s EEG response to body and non-body (house) stimuli. Reaction time and accuracy were also monitored in order to ascertain whether potential ERP effects were related to behaviour.

Previous research has found the amplitudes of early face selective components, error-related components and both early and late visual and auditory components to be highly reliable, whilst their latencies appear to be less reliable [26–28, 39–42]. Reliable ERP responses to emotional face stimuli within individuals have also been reported, as one study found no changes in N170, VPP, medial frontal negativity (MFN), feedback-related negativity (FRN), P3, and late positive potential (LPP) amplitude over a 4-week period [26]. Also, it has been reported that face-selective ERP responses are stable at 4 years, 17 years and adulthood, as P1, N170 and N250 amplitude, latency and topography presented similarly in all three age groups [43]. Although body perception is understood to be distinct from face perception [3, 10, 16, 17, 44], the underlying processes are thought to be similar [9]. Therefore, in line with previous research, we predicted that early visual ERP amplitudes and body-sensitive effects (i.e. amplitude differences between body and non-body stimuli) would be reliable, whereas early visual ERP latencies may be less reliable. Finding stable ERPs and body-sensitive effects would suggest that these electrophysiological responses could potentially serve as neuropsychiatric biomarkers.

## Materials and methods

### Participant information

Seven men and seven women from the University of Essex were recruited to participate. Demographic information such as age, exercise habits and area of work and/or study were collected in order to determine whether participants were primed by their lifestyle to recognise or focus on bodies. The average age of male participants was 26 years (SD 3.45 years) and of female participants was 31 years (SD 6.03 years). The average amount of exercise per week was approximately 7 hours (SD 2.65 hours) for men, and 6.5 hours (SD 4.76 hours) for women. For all participants this was generally aerobic in nature. One man and one woman reported an area of study with particular focus on the body: Sports Science. Participants were financially reimbursed for their time.

### Exclusion criteria

Those who reported a clinical history of body perception disorders, eating disorders, or a major psychiatric disorder such as schizophrenia or bipolar disorder, were not permitted to take part.

One male participant failed to return after the first session so his data were not included. During the study, another participant disclosed a transgender identity and informed us that they were beginning the gender reassignment procedure. We felt the conflict between the physical sex of the body and gender identity could be a potential confound to the requirements of the task and thus excluded these data. Therefore, data were analysed from six men and six women who completed the full 4-week test-retest protocol.

### Ethical declaration

The study was conducted in line with the 2008 Declaration of Helsinki, and was approved by the Ethics Committee for the Department of Psychology at the University of Essex. Informed written consent was obtained from each participant before the study commenced and all participants consented to photographs of their body (without the head) being used as stimuli for other participants in this study.

### Apparatus and stimuli

#### Questionnaire

A ‘week-to-week comparison’ questionnaire was also devised and administered for the purpose of this study. It consisted of self-report questions designed to probe how focused, stressed, tired and hungry the participants were from one session to the next. It also assessed caffeine and medication intake whilst giving the opportunity for participants to report ‘any other information’ that they thought may affect their performance. This was done in order to assess consistency between sessions and also to act as a reminder to the participant about the importance of controlling as many of these factors as possible from week to week.

#### EEG stimuli

Each participant saw images of houses, their own body and other participants’ bodies. Responses to own-body stimuli were collected in line with hypotheses relevant to another research project. In particular, we were interested in a potential modulation of body-sensitive P1, N1 and VPP components in response to own-body viewing.

Using a Minolta Dimage A2 camera, participants were photographed standing, sitting and lying in white vest top and briefs that were provided by the experimenter. This photoshoot was conducted against a black background that was lit by two 3x36W fluorescent units, with a cool white colour temperature of 4100K. Images were edited using Adobe PhotoShop to ensure backgrounds were completely black and the head was cropped.

In total, 11 pictures of each participant’s full body, without the head, were used. These included 3 standing, 3 sitting and 3 lying down, with views of each position from the front, side and back. There were also 2 kneeling positions included; 1 to the front and 1 to the side. In addition, 16 pictures of each participant’s body parts were used. These depicted varying viewpoints and included 3 of the extended torso (neck to mid-thigh), 2 of the stomach, 1 of the chest, 1 of the torso (neck to hips), 2 of the leg bent at the knee, 3 of the whole legs and 4 of the whole arm.

**Fig 1:**
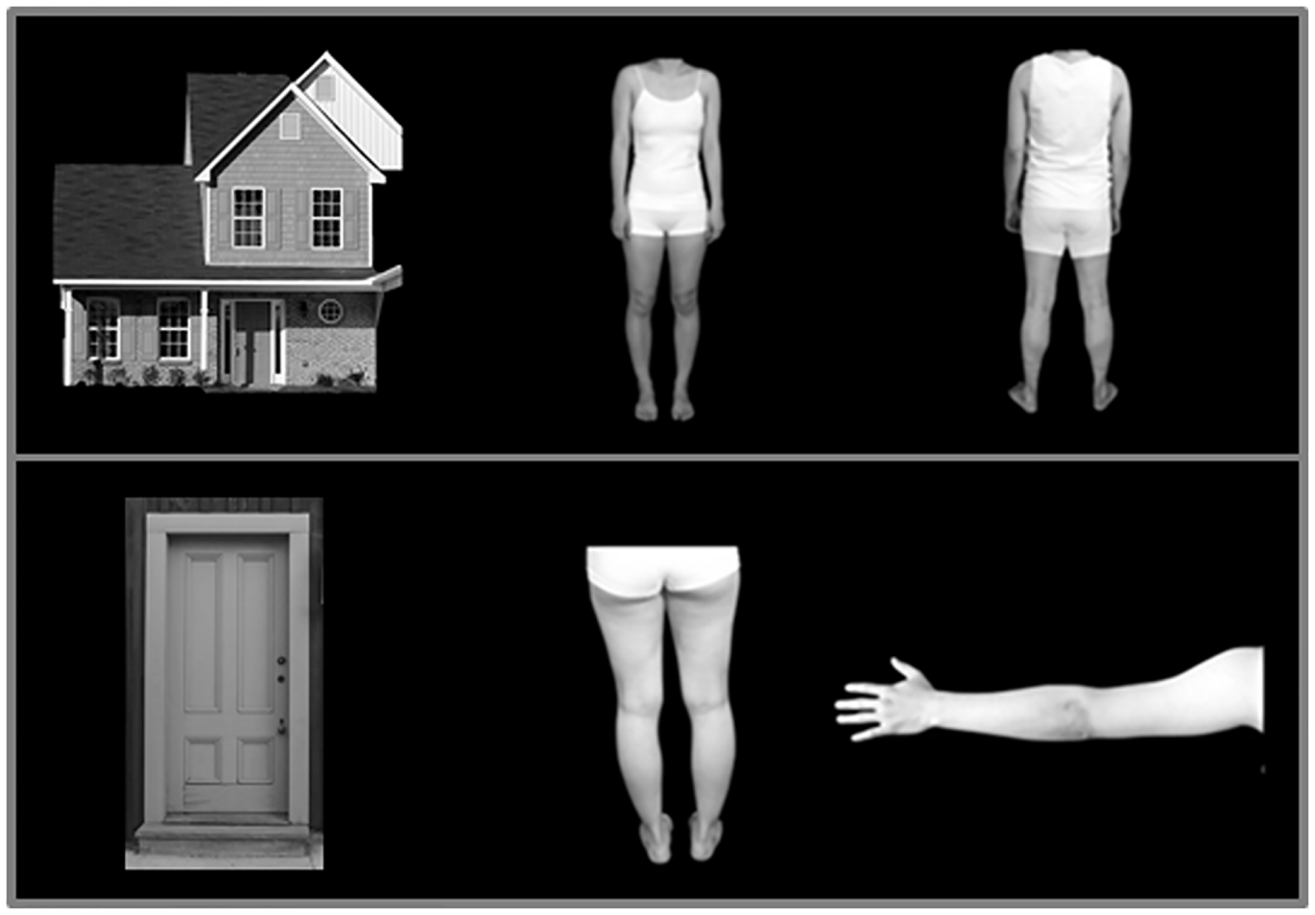
Example stimuli controlled for overall image brightness. (Top: whole stimuli, bottom: parts of stimuli, far left panel: house stimuli, middle panel: female body stimuli, far right panel; male body stimuli).

Two stimulus sets were created, one for male participants and one for female participants, whereby ‘other’ body stimuli were images of the ‘other’ participants of the same gender. Thus, a set of 162 ‘other-body’ stimuli and 27 ‘own-body’ stimuli, was created for each participant (see Fig 1 for examples).

In addition, 66 whole-house images of varying viewpoints and 96 images of house parts were downloaded from the Worldwide Web; this included 14 conservatories, 14 doors, 14 porches, 14 roofs, 14 windows, 13 images of the top half of a house and 13 images of the bottom half of a house (see Fig 1).

All stimuli were 1024 pixels in width and 768 pixels in height and had their brightness adjusted to control for luminance across all images.

### Procedure

Participants were asked to individually attend five sessions; one photo session, and four EEG test sessions that ran at the same time for four consecutive weeks. Prior to participation, each individual was fully briefed on the procedure of each session via email.

#### Photograph session

The duration of the photo session was one hour. Upon arrival, participants were given written instructions and the opportunity to ask questions before written consent was obtained. Following this, each participant was shown to a private booth where they changed into a white vest top and white briefs provided by the experimenter. Sizes had been requested in advance. All jewellery was removed and long hair was tied back. Those who requested to wear white underwear underneath the clothing provided were permitted to.

Participants were directed by the experimenter to pose in the centre of the photographic set-up. Each participant was given the option to view the images and delete those they did not find satisfactory before leaving the session. None did so.

#### Test sessions

Participants returned for the first test session from between 1 week to 1 month after the photograph session. A standardised overview of procedures was read and written consent was obtained at the start of each session. The week-to-week questionnaire was completed during EEG preparation.

Participants were seated approximately 60 cm from the screen to complete an identification task in which they had to decide whether the image shown was of their own body, another body or a house. Emphasis was placed on speed and accuracy. They were asked to fixate on the centre of the screen whereby a white cross was presented for 250 ms on a black background. Stimuli were then shown for 250 ms, followed by a 1500 ms response interval that terminated once a response was given. A random intertrial interval of between 300 ms and 700 ms separated individual trials.

Participants were instructed to give one of three responses by pressing either the ‘O’ key to ‘other’ with the right index finger, the ‘S’ key to ‘self’ with the left index finger, or the space bar to ‘house’ with both thumbs.

Own-body stimuli were shown 6 times, ‘other’ body stimuli taken from the 6 other participants of the same gender and house stimuli were shown once. Thus, there were 486 trials in total, separated into 12 blocks of 40 trials and a final block of 6 trials. The timing between blocks was at the participant’s discretion. Stimuli were randomised with a cumulative summary of detection times and errors displayed during inter-block intervals.

### ERP/EEG recording

#### EEG acquisition

Continuous EEG was sampled at a rate of 500 Hz from 64 Ag/AgCl electrodes placed according to the international 10–10 system (EASYCAP GmbH, Herrsching, Germany). Online, the signal was referenced to the left earlobe with impedances kept below 10 kΩ. Signals from the right earlobe were also recorded. Bipolar channels recorded vertical (VEOG) and horizontal (HEOG) electro-oculogram from above and below the midpoint of the right eye and beside the outer canthi of both eyes. Recording and offline analysis of EEG and EOG data was conducted with Neuroscan Synamps2 system and SCAN 4.5 software (Compumedics, Melbourne, Australia).

Offline, EEG and EOG signal were digitally filtered using a 30-Hz lowpass filter with 24 dB slope, then re-referenced to the average of the left and right earlobes.

#### Segmentation

The data were divided into 600 ms epochs beginning 100 ms prior to stimulus onset and baseline corrected against the mean voltage during the 100 ms pre-stimulus period.

#### Artifact detection

Trials with horizontal eye movements (HEOG exceeding ± 50 μV relative to baseline), eye blinks or other artefacts (a voltage exceeding ± 100μV at any electrode relative to baseline) were rejected from further analysis.

### Statistical analyses

Because we expected ‘own-body’ viewing to modulate early visual body processing, [7, 45–50], and in order to avoid any potential confounding effects, responses to ‘own body’ stimuli were removed from both behavioural and ERP analyses. That is, only trials in which others’ bodies and houses were shown were entered into analyses. This is also in keeping with other studies of early visual body processing, [10].

To allow for clearer inferences about the probabilities of both significant and nonsignificant effects in our data, Bayesian probabilities associated with the occurrence of both the null hypothesis (H_0_|D) and the experimental hypothesis (H_1_|D) were calculated alongside standard statistics [51]. These probabilities range from 0 (no evidence) to 1 (very strong evidence).

#### Behavioural analyses

Accuracy and reaction time data were subjected to a separate 2 x 4 (picture type vs. week) repeated measures ANOVA in order to assess how accurate and how fast participants were at identifying body and house stimuli across the weeks.

#### Electrophysiology

ERP waveforms were averaged across the viewing conditions to be included in analyses (other body stimuli and house stimuli) and the average number of rejected trials per condition can be viewed in Table 1.

**Table 1:** Average number of rejected trials per condition each week (n = 12), rounded to the nearest integer, out of 162 trials. Standard deviations are in parentheses.

	Week 1	Week 2	Week 3	Week 4
Bodies	40 (29)	28 (25)	28 (20)	23 (21)
Houses	40 (32)	27 (22)	34 (35)	26 (20)

**Fig 2:**
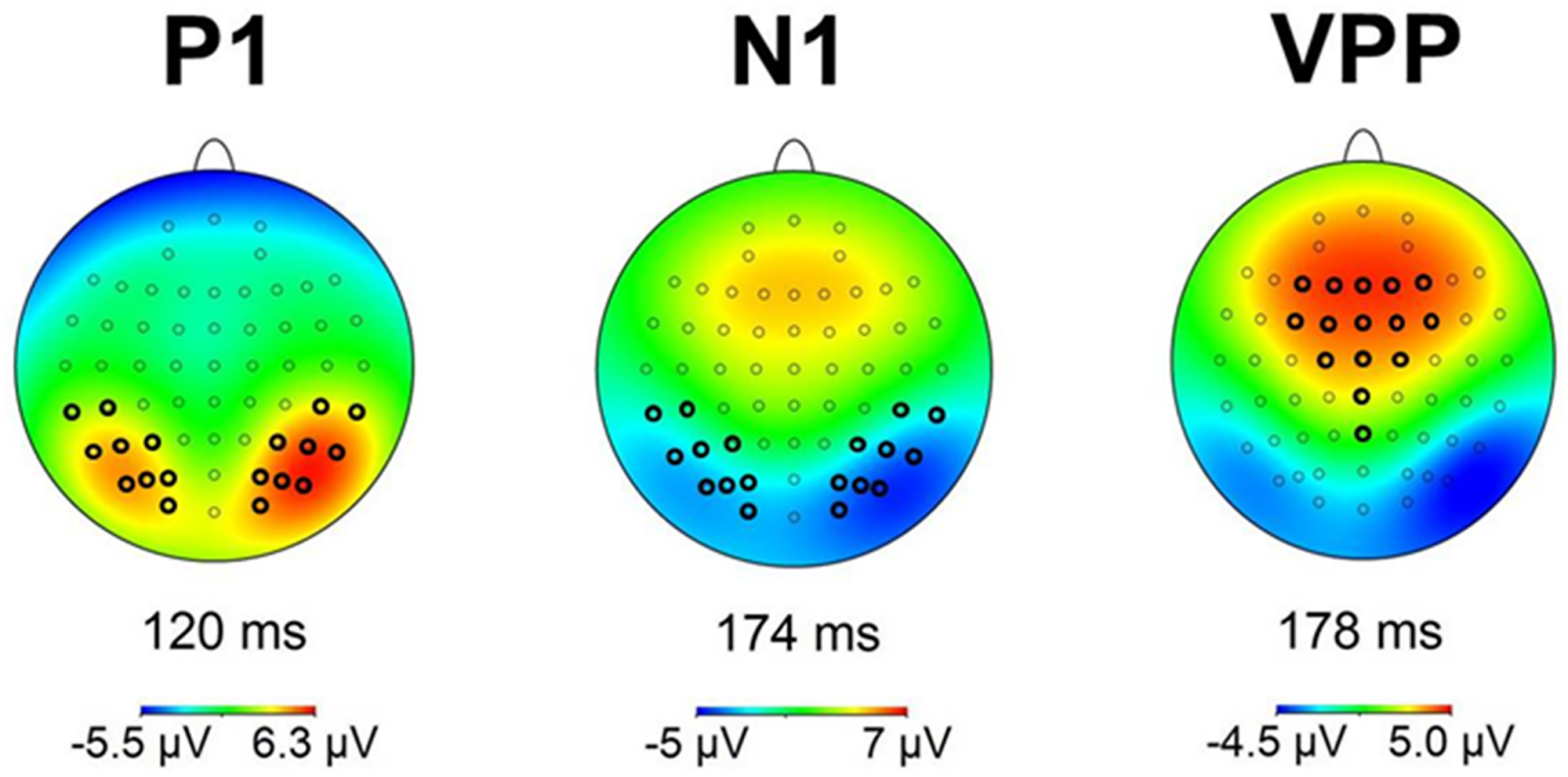
Voltage maps for the time window of the visual P1 component, visual N1 component and visual VPP component peaks (P1 at 120 ms, N1 at 174 ms, VPP at 178 ms), collapsed over viewing conditions, confirming areas of strongest activation. Electrodes analysed, which were selected primarily based on previous literature, have been highlighted.

In order to assess the reliability of early visual ERPs and body-sensitive processing, peak amplitude and peak latency data were analysed in P1, N1 and VPP time ranges (time windows determined on the basis of the aggregated grand average, P1: 100 ms– 130 ms, N1: 155 ms– 195 ms, VPP: 155 ms– 195 ms) at all electrodes previously implicated in body processing. These included *O1/2*, *PO3/4*, *PO5/6*, *PO7/8*, *P7/8*, *P5/6*, *TP7/8* and *CP5/6* for P1 and N1 [10, 11, 18, 19, 23, 52–54] and *F1/2*, *F3/4*, *FC1/2*, *FC3/4*, *C1/2*, *Fz*, *Fcz*, *Cz*, *CPz* and *Pz* for the VPP [16, 18, 19, 55]. Topographic maps of individual visual components in the current sample confirmed this selection for occipito-parietal electrodes, whilst suggesting VPP activity might not be as posterior as has been found in other studies (see Fig 2).

ERPs to body and house stimuli were compared to assess for selective responses to bodies over occipito-parietal (P1 and N1 components) and fronto-central (VPP component) electrodes. Body-sensitive effects and overall amplitudes and latencies of these components were compared between weeks to assess whether early visual components and/or body processing effects were stable over time. Reliability analyses thus addressed two questions; first, whether early visual components are stable and second, whether body-sensitive effects are stable. Consequently, both amplitude and latency data for each component were subjected to a repeated measures ANOVA with week (week 1 vs. week 2 vs. week 3 vs. week 4), picture type (houses vs. bodies) hemisphere (left vs. right for P1/N1 analyses only) and electrode (as above) as within-subjects factors. To get a comprehensive idea of the robustness of each ERP at individual electrode sites, amplitudes and latencies of each component were subject to Pearson’s product-moment correlations between weeks, separately at each electrode. To quantify the reliability of components and body-sensitive effects, between-week robustness was further compared to within-week robustness between nearby electrodes.

## Results

### Behavioural results

#### Accuracy analyses

**Fig 3:**
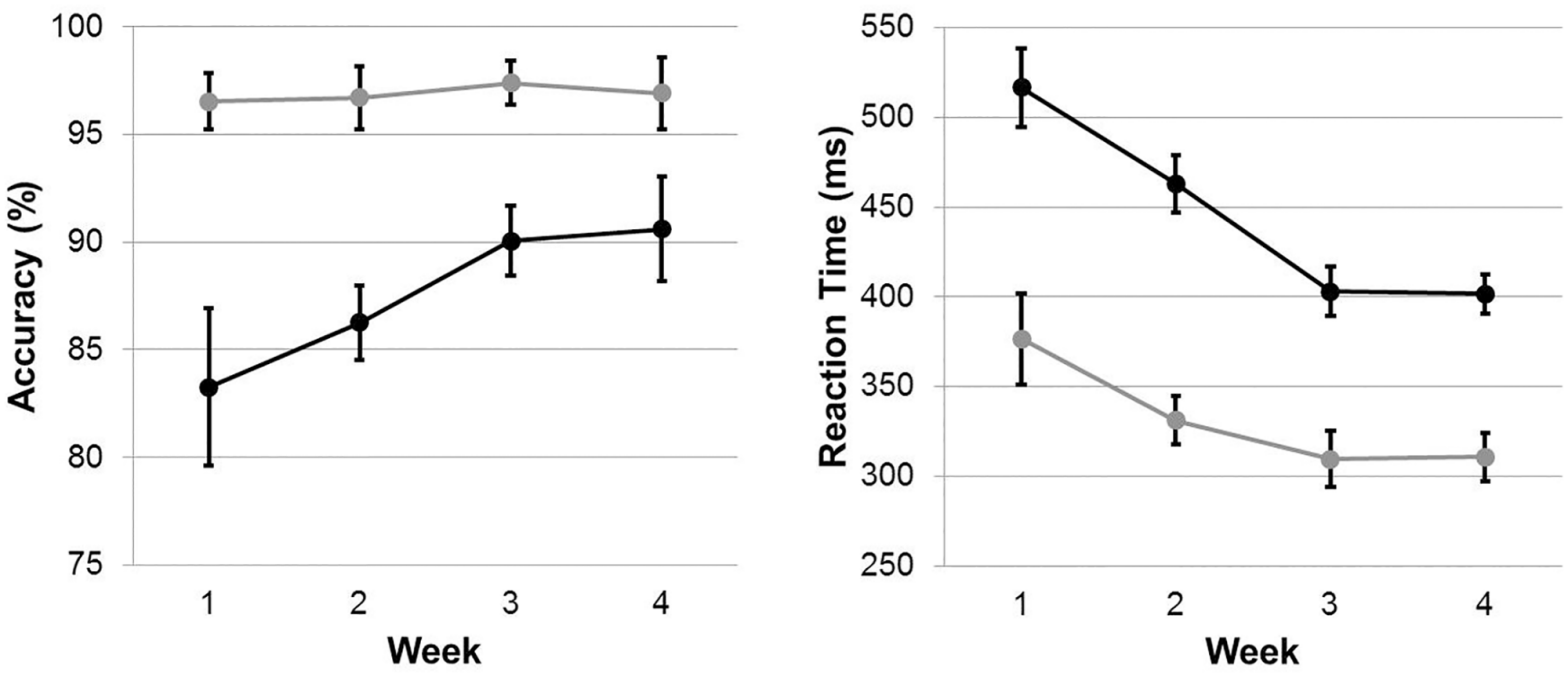
Left panel shows task accuracy in percent over the 4 testing weeks. Participants were more accurate to identify house stimuli (grey line) than body stimuli (black line). Right panel shows reaction time in ms over the four testing weeks, which systematically decreased. Participants responded faster to houses (grey line) than to body stimuli (black line).

A 2 x 4 (picture type vs. week) repeated measures ANOVA revealed a main effect of picture type (*F*(1, 11) = 17.72 *p* = .001, *η*_*p*_^2^ = .62, p(H_1_|D)>.99) as participants responded to houses more accurately than bodies each week, and a main effect of week (*F*(3, 33) = 4.59, *p* = .033, *η*_*p*_^2^ = .29, p(H_1_|D)>.71) as accuracy increased over the weeks (see Fig 3). These main effects were qualified by a significant two-way interaction between the factors (*F*(3, 33) = 5.99, *p* = .005, *η*_*p*_^2^ = .35, p(H_1_|D)>.92). Bonferroni corrected follow-up comparisons showed that accuracy did not differ between the weeks in response to houses (*t*(11) ≤ .843, *p* = 1.00), whilst there was a significant increase in accuracy to body stimuli between weeks 2 and 3 (*t*(11) = 4.03, *p* = .012). No other differences were found (see Fig 3).

#### Reaction time analyses

A 2 x 4 (picture type vs. week) repeated measures ANOVA also revealed a main effect of picture type (*F*(1, 11) = 45.07, *p* < .001, *η*_*p*_^2^ = .80, p(H_1_|D) >.99) as participants responded to houses more quickly than bodies each week (see Fig 3) and a main effect of week (*F*(3, 33) = 29.71, *p* < .001, *η*_*p*_^2^ = .73, p(H_1_|D)>.99) as RT decreased as the weeks progressed. The two main effects were qualified by a significant two-way interaction (*F*(3, 33) = 7.86, *p* = .003, *η*_*p*_^2^ = .42, p(H_1_|D)>.99). As suggested by Fig 3, Bonferroni adjusted follow-up comparisons found that in response to bodies, performance improved between weeks 1, 2 and 3 (*t*(11) ≥ 3.59, *p* < .025) but did not differ then between weeks 3 and 4 (*t*(11) = .20, *p* = 1.00). In response to houses however, performance only differed between weeks 1 and 3 (*t*(11) = 3.59, *p* = .026) such that RT was quicker in week 3 (309 ms) compared to week 1 (376 ms).

### ERP results

Both peak amplitude and peak latency data for each component were subjected to a repeated measures ANOVA with week (week 1 vs. week 2 vs. week 3 vs. week 4), picture type (houses vs. bodies) hemisphere (left vs. right for P1/N1 analyses only) and electrode (as above) as within-subjects factors.

#### P1 peak amplitude

**Fig 4:**
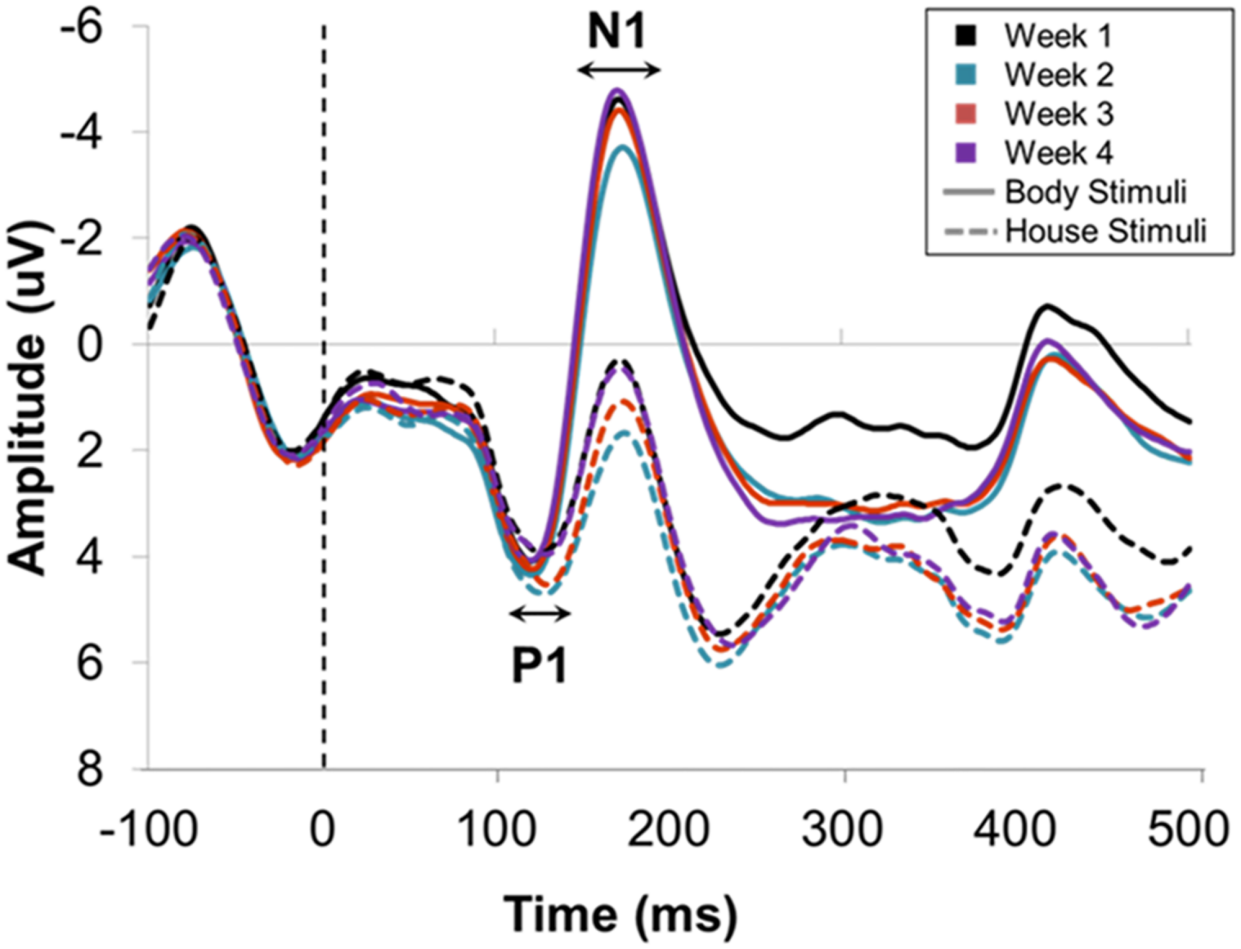
Grand averaged ERP responses during house and body viewing over the four weeks (bodies with solid lines, houses with dotted lines and week 1 to week 4 colour coded) collapsed over electrodes O1/2, PO3/4, PO5/6, PO7/8, P7/8, P5/6, TP7/8, CP5/6. A body-sensitive N1 response is evident each week, whilst amplitudes and latencies for both components were not found to differ between weeks.

P1 amplitudes were found to be stable over time as ANOVA found no effect of week (*F*(3, 36) = .90, *p* = .449, *η*_*p*_^2^ = .07, p(H_1_|D) < .01). Furthermore, amplitudes were not modulated by picture type (*F*(1, 12) = .03, *p* = .856, *η*_*p*_^2^ < .01, p(H_1_|D) < .23), which was a stable finding as this did not interact with week (*F*(3, 36) = 1.61, *p* = .207, *η*_*p*_^2^ = .12, p(H_1_|D) < .04). Picture type did interact with electrode however, (*F*(7, 84) = 10.13, *p* = .003, *η*_*p*_^2^ = .46, p(H_1_|D)>.99), with Bonferroni-adjusted follow-up comparisons revealing larger amplitudes to houses (M ≥ 3.59 μV) than to bodies (M ≥ 2.81 μV) at electrodes TP7/8, CP5/6, P5/6 (*F*(1, 12) ≥ 5.61, *p* ≤.036, *η*_*p*_^2^ ≥ .32), whilst at O1/2 amplitudes were larger to bodies (M = 5.86) than to houses (M = 3.75) (*F*(1, 12) = 5.71, *p* = .034, *η*_*p*_^2^ = .32). This suggests that there may be some distinction between bodies and other stimuli as early as the P1 time range over some electrode sites (see Fig 4). Hemispheric differences were also found, (*F*(1, 12) = 8.67, *p* = .012, *η*_*p*_^2^ = .42, p(H_1_|D)>.88), as amplitudes were larger in the right hemisphere (5.84 μV) compared to the left hemisphere (4.75 μV). In sum, P1 amplitudes are seemingly stable, are larger in the right hemisphere, and differentiate between bodies and other stimuli over some electrodes only.

#### P1 peak latency

P1 latencies were found to be stable over time as ANOVA revealed no effect of week (*F*(3, 33) = .05, *p* = .920, *η*_*p*_^2^ < .01, p(H_1_|D) < .01). Body sensitive effects were not observed on P1 latency (*F*(1, 11) = 2.33, *p* = .155, *η*_*p*_^2^ = .18, p(H_1_|D) < .48), which was a stable finding as there was no interaction with week (*F*(3, 33) = 1.02, *p* = .380, *η*_*p*_^2^ = .09, p(H_1_|D) < .02) (see Fig 4). Picture type did interact with electrode however, (*F*(7, 77) = 14.70, *p* < .001, *η*_*p*_^2^ = .58, p(H_1_|D) = 1) with faster responses to bodies compared to houses at more lateral sites TP7/8, CP5/6, P7/8 and P5/6 (*F*(1, 11) ≥ 7.86, *p* ≤.017, *η*_*p*_^2^ ≥ .42). Accordingly, P1 latencies appear to be stable and there may be some distinction between bodies and other stimuli over specific electrode sites.

#### N1 peak amplitude

ANOVA revealed that N1 amplitudes were stable over time as no effect of week was found (*F*(3, 36) = 1.69, *p* = .210, *η*_*p*_^2^ = .12, p(H_1_|D) < .04). Body-sensitivity was identified as a main effect of picture type (*F*(1, 11) = 47.98, *p* < .001, *η*_*p*_^2^ = .80, p(H_1_|D)>.99), such that amplitudes were more negative to bodies (M = -5.45 μV) in comparison to houses (M = -.22 μV). This was a stable finding as there was no interaction between picture type and week (*F*(3, 36) = .56, *p* = .624, *η*_*p*_^2^ = .05, p(H_1_|D) < .01) (see Fig 4). Picture type was found to interact with electrode however (*F*(7, 84) = 13.75, *p* < .001, *η*_*p*_^2^ = .53, p(H_1_|D)>.99). Follow-up comparisons revealed body-sensitive effects at all electrode sites (*F*(1, 12) ≥ 16.24, *p* ≤.002, *η*_*p*_^2^ ≥ .58) but mean differences (*MD*) appeared to be larger at more posterior sites (*P7/8*, *P5/6*, *PO7/8*, *PO5/6 MD* ≥ 6.39 μV) compared to lateral and occipital sites (*TP7/8*, *CP5/6*, *PO3/4*, *O1/2 MD* ≤ 4.29 μV). Thus, N1 amplitudes appear to be stable and reliably body-sensitive.

#### N1 peak latency

ANOVA found N1 peak latencies to be stable over time as there was no effect of week (*F*(3, 33) = 1.69, *p* = .214, *η*_*p*_^2^ = .13, p(H_1_|D) < .05). There was also no difference in latency between body viewing and house viewing (*F*(1, 11) = .43, *p* = .524, *η*_*p*_^2^ = .04, p(H_1_|D) < .27), which was a stable finding as this did not interact with week (*F*(3, 33) = 2.88, *p* = .072, *η*_*p*_^2^ = .21, p(H_1_|D) < .23). Picture type was found to interact with electrode however, (*F*(7, 77) = 5.19, *p* < .013, *η*_*p*_^2^ = .32, p(H_1_|D)>.67). Follow-up comparisons revealed no differences between body and house viewing at any of the electrode sites (*F*(1, 12) ≤ 3.92, *p* ≥ .073, *η*_*p*_^2^ ≤ .26), although *MD* appeared to be smaller at posterior sites (*P7/8*, *P5/6 MD* ≤ .34 ms) compared to temporal (*TP7/8*, *CP5/6 MD* ≥ 1.42 ms) or occipito-parietal sites (*PO7/8*, *PO5/6*, *PO3/4*, *O1/2 MD* ≥ 2.15 ms). Nonetheless, in sum, N1 latencies appear to be stable over time without differentiating between bodies and other stimuli (see Fig 4).

#### VPP peak amplitude

**Fig 5:**
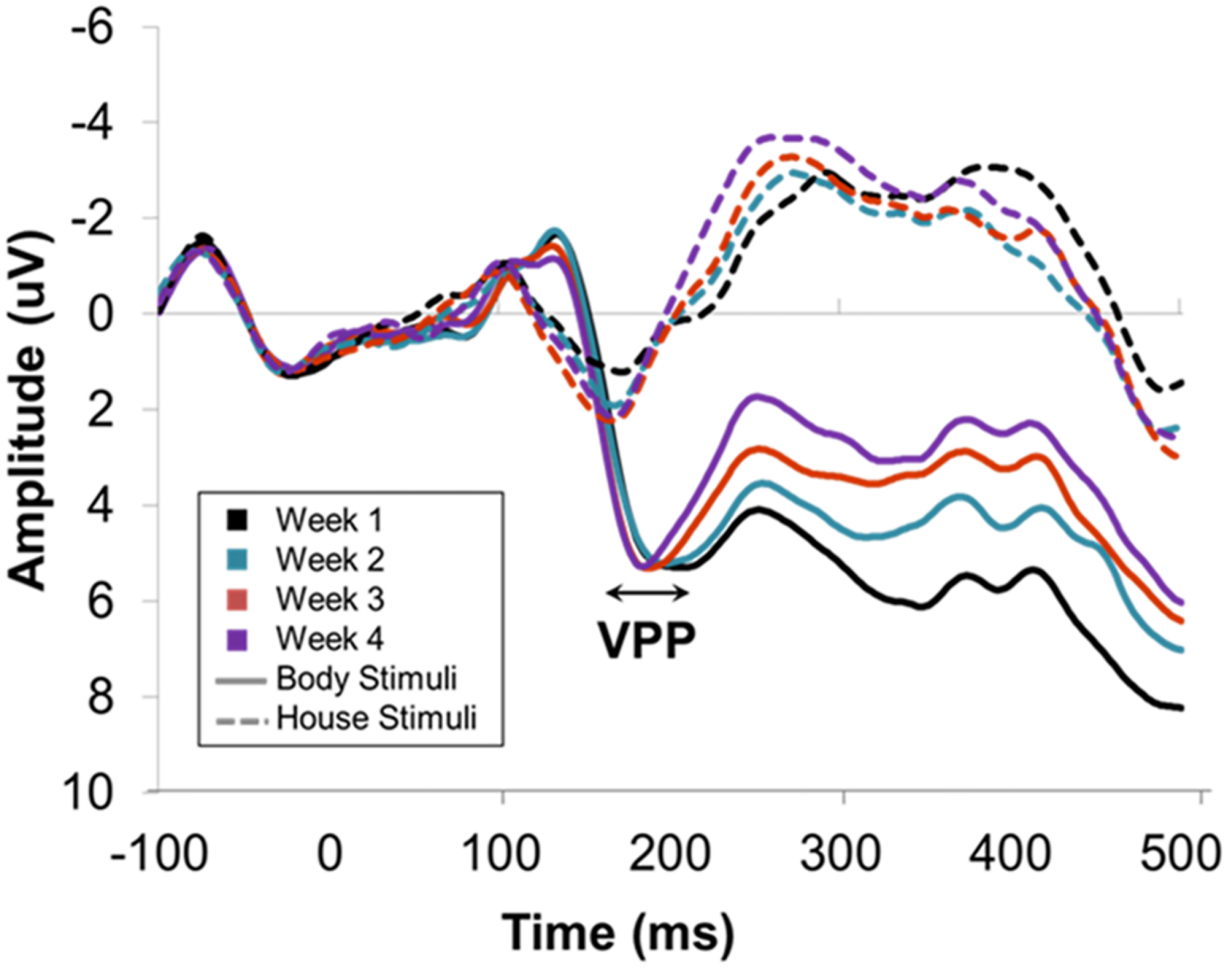
Grand averaged ERP responses during house and body viewing over the four weeks (bodies with solid lines, houses with dotted lines and week 1 to week 4 colour coded as above) collapsed over electrodes F1/2, F3/4, FC1/2, FC3/4, C1/2, Fz, Fcz, Cz, CPz and Pz. A body-sensitive VPP response is evident each week, whilst amplitudes and latencies were not found to differ between weeks.

ANOVA found VPP amplitudes were stable over time as there was no effect of week (*F*(3, 33) = 1.37, *p* = .276, *η*_*p*_^2^ = .11, p(H_1_|D) < .04). A body-sensitive effect was found (see Fig 5), such that amplitudes were more positive to bodies (M = 6.48 μV) in comparison to houses (M = 3.20 μV) (main effect of picture type: *F*(1, 11) = 19.58, *p* = .001, *η*_*p*_^2^ = .64, p(H_1_|D)>.99). This effect was also stable as picture type was not found to interact with week (*F*(3, 33) = .78, *p* = .471, *η*_*p*_^2^ = .07, p(H_1_|D) < .02) (see Fig 5). A picture type by electrode interaction was also found (*F*(14, 154) = 24.61, *p* < .001, *η*_*p*_^2^ = .69, p(H_1_|D) = 1), with Bonferroni-adjusted follow-up comparisons revealing body-sensitive effects at all electrodes (*F*(1, 11) ≥ 10.77, *p* ≤ .007, *η*_*p*_^2^ ≥ .50) except *CPz* and *Pz* (*F*(1, 11) ≤ 4.36, *p* ≥.061, *η*_*p*_^2^ ≥ .28). These findings therefore suggest that VPP amplitudes and body-sensitive effects over the VPP are stable, although such body sensitivity might not occur over more posterior regions in all studies.

#### VPP peak latency

VPP latencies were found to be stable as ANOVA revealed no effect of week (*F*(3, 33) = 2.92, *p* = .073, *η*_*p*_^2^ = .21, p(H_1_|D) < .24). A main effect of picture type was found (*F*(1, 11) = 13.08, *p* = .004, *η*_*p*_^2^ = .54, p(H_1_|D)>.97), such that VPP responses were shorter to houses (M = 169 ms) in comparison to bodies (M = 185 ms) (see Fig 5). This effect was also stable as there was no interaction between picture type and week (*F*(3, 33) = 1.59, *p* = .224, *η*_*p*_^2^ = .13, p(H_1_|D) < .05) (see Fig 5). A significant picture type by electrode interaction was found, however (*F*(14, 154) = 7.33, *p* < .001, *η*_*p*_^2^ = .40, p(H_1_|D)>.99), with Bonferroni-adjusted follow-up comparisons revealing differences in VPP latency between bodies and houses at all electrodes (*F*(1, 11) ≥ 5.07, *p* ≤.046, *η*_*p*_^2^ ≥ .32) except Pz (*F*(1, 11) = .08, *p* = .781, *η*_*p*_^2^ = .01). This further suggests that body sensitivity over VPP may occur at more anterior sites. In sum, there appears to be a distinction between bodies and other stimuli evident in VPP latencies, which, like VPP latency itself, is a seemingly stable finding.

### Interim summary of reliability analyses

Amplitudes and latencies of early visual components over occipito-parietal (P1, N1) and fronto-central (VPP) sites appear to be stable over time. Body-sensitive effects measured here (for some electrodes over P1, most electrodes over VPP, and all electrodes over N1) also appear to be stable.

### Assessing the robustness of early visual ERPs and their associated body-sensitive effects; the relationship between weeks at each electrode site

#### Visual components

**Fig 6:**
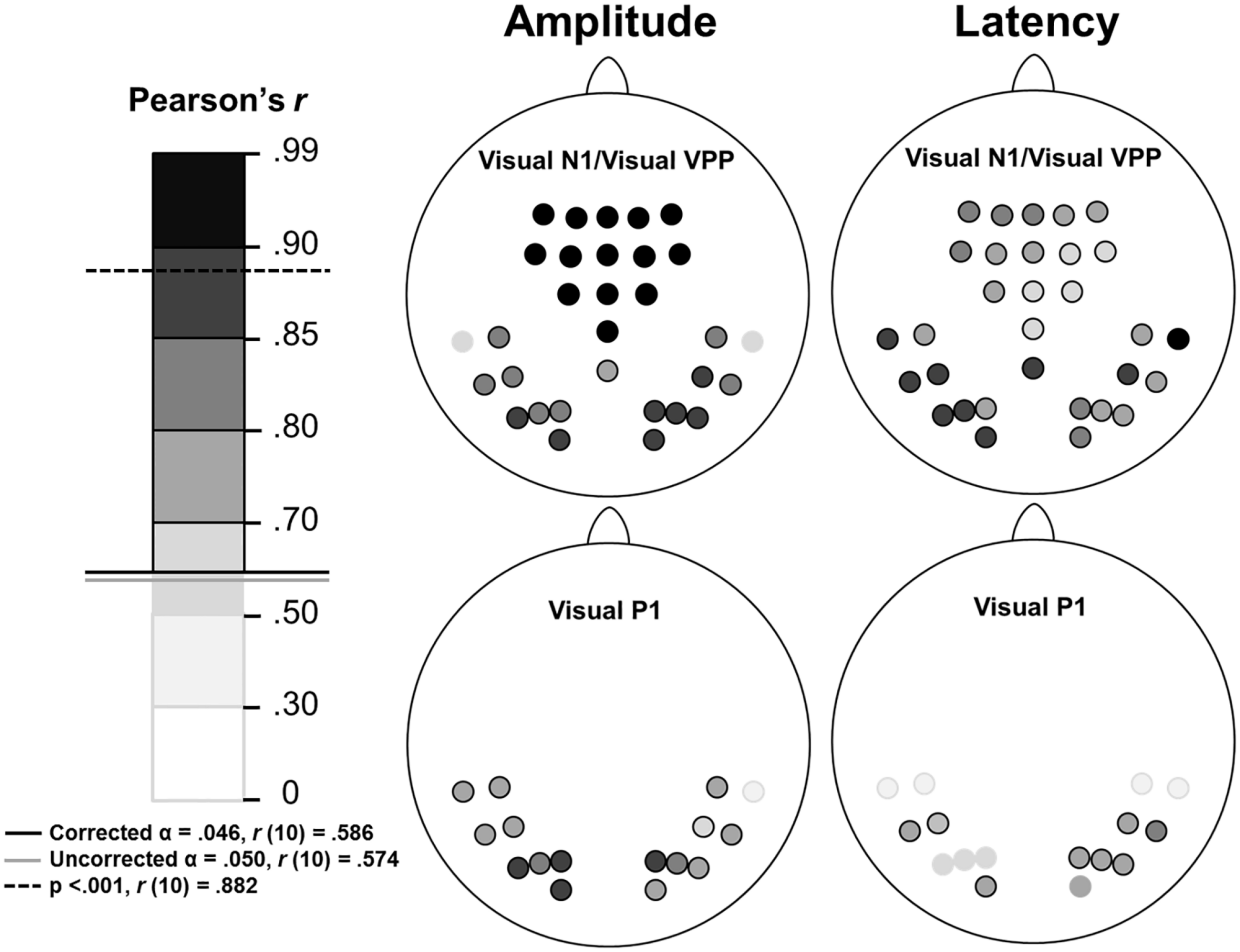
Visual ERP amplitude and latency correlations between the weeks at each analysed electrode site are depicted separately above. For each electrode site the weakest correlation (lowest Pearson’s r-value) is depicted out of the six possible combinations of the four weeks. Left panel shows the r-value scale: darker colours indicate stronger relationships. Shades above the solid black line were significant after FDR correction (α = .046). Right panel shows scalp maps of analysed electrodes for each component (posterior electrodes for visual P1/N1 and anterior electrodes for visual VPP) for both amplitude and latency. Where all relationships between all weeks were significant the electrode is outlined in black. When at least one non-significant relationship was found between two of the weeks the electrode is outlined in grey.

The above analyses demonstrate that early visual amplitudes and latencies are stable over time at the group level. Nevertheless, those analyses do not rule out the possibility that some individuals show poor reliability over time, but that this is obscured by the group-level analysis (i.e., there is no consistent time-dependence across the group to reach statistical significance). To investigate this possibility, and to further assess the reliability of these early visual ERPs, amplitudes and latencies for each week were averaged across conditions at each electrode site used in the P1, N1 and VPP ANOVAs. Both amplitude and latency data for each week were then subjected to separate Pearson’s product-moment correlational analyses at each electrode. Six relationships between the weeks, representing each possible pairing of weeks, were therefore obtained at each electrode site separately for amplitude and latency data, and are summarised below (see Fig 6). In order to compare the reliability, across weeks, of latency versus amplitude, *r*-values representing all six bivariate between-week correlations were averaged for each electrode and entered into pairwise *t*-tests. These test revealed that, frontally, correlations of VPP amplitudes across time were larger than correlations of VPP latencies (*t*(14) = 6.82, p < .001) suggesting that VPP amplitude is more reliable that VPP latency. However, posteriorly, analogous correlations across time for amplitudes were not significantly different from correlations across time for latencies for either P1 (*t*(14) = 1.98, p = .068) or N1 (*t*(14) = .60, p = .56).

**Fig 7:**
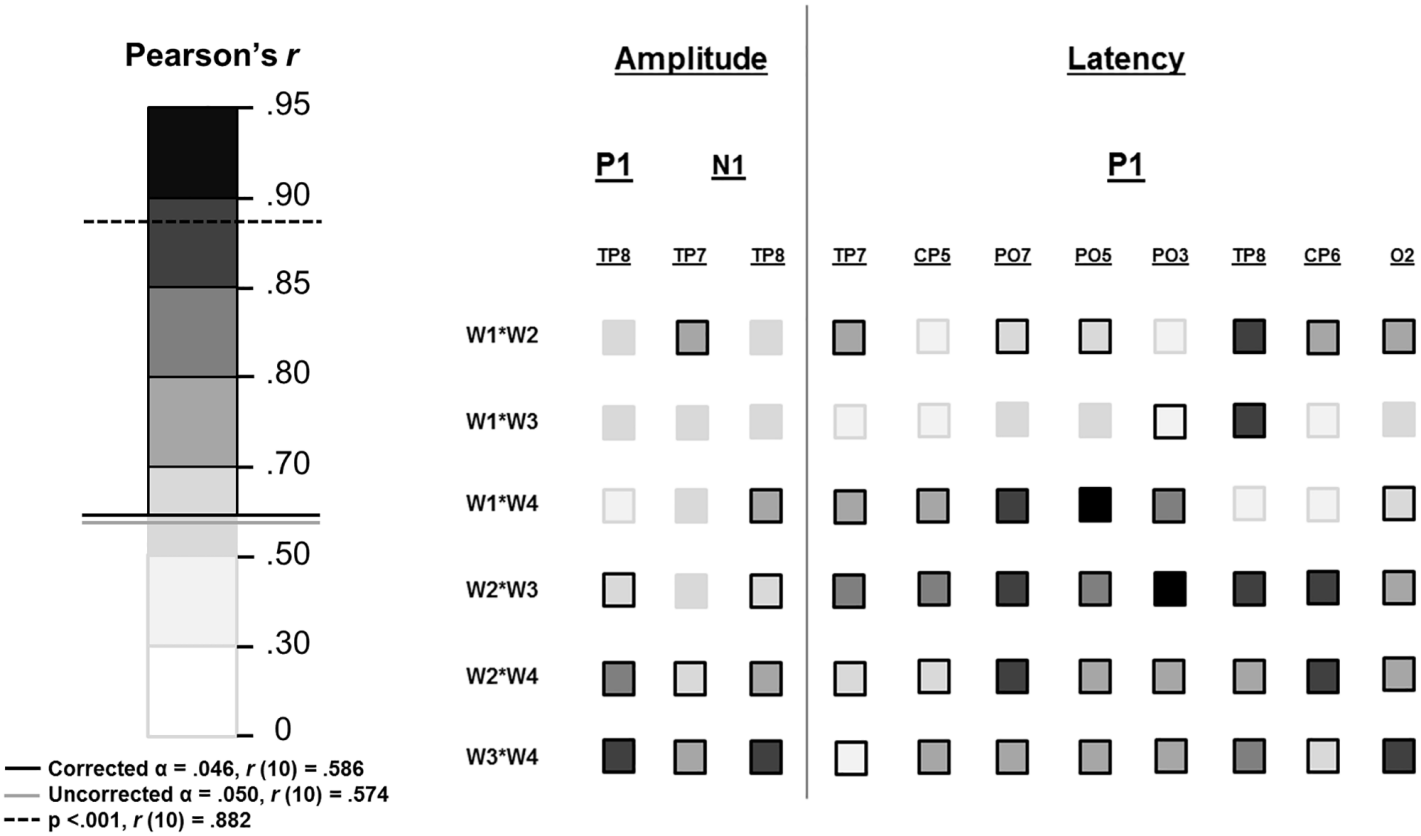
Visual ERP amplitude and latency correlations between weeks at electrode sites that did not yield six significant relationships. Left panel shows the scale pertaining to Pearson’s r values, with darker colours indicative of a stronger relationship. Relationships indicated by a shade above the solid black line were significant after FDR correction (α = .046). Right panel shows separate week-to-week correlations at electrode sites where not all relationships were significant for P1 amplitude and P1, N1 and VPP latency. Non-significant relationships are outlined in grey; significant relationships are outlined in black.

For electrode sites that did not yield six significant relationships, the pairings are listed separately in Fig 7. Upon viewing this, it is clear that early posterior amplitude and latency are less robust at lateral sites. At least for studies that utilise body stimuli, this suggests that occipito-parietal, rather than more temporal, sites may be a more reliable source of the P1-N1 complex.

#### Body-sensitive effect

**Fig 8:**
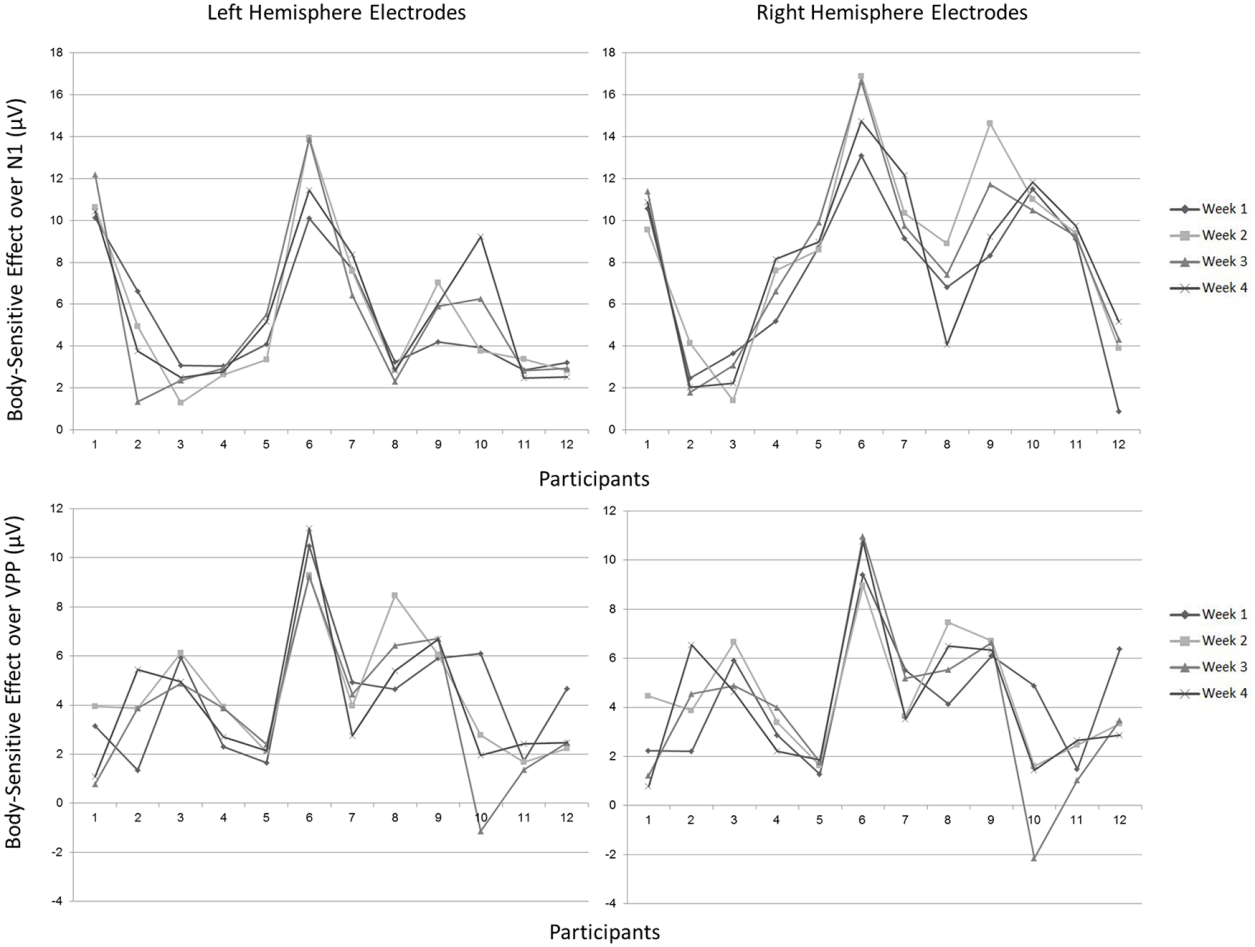
Body-sensitive effects for each participant (1–12), each week, over electrodes P7/8 for N1 and F1/2 for VPP. The left panel shows left hemisphere electrodes, whilst the right panel shows right hemisphere electrodes. The top panel shows body-sensitivity over N1, whilst the bottom panel shows body-sensitivity over VPP.

In order to further assess the reliability of body-sensitive ERP effects, the difference in amplitude between body and house viewing was calculated at each electrode site, for each week, for both N1 and VPP components. Fig 8 illustrates these body-sensitive ERP effects for each participant, each week, over electrodes sites where maximal responses were recorded. This was over P7/8 for N1 and F1/2 for VPP.

**Fig 9:**
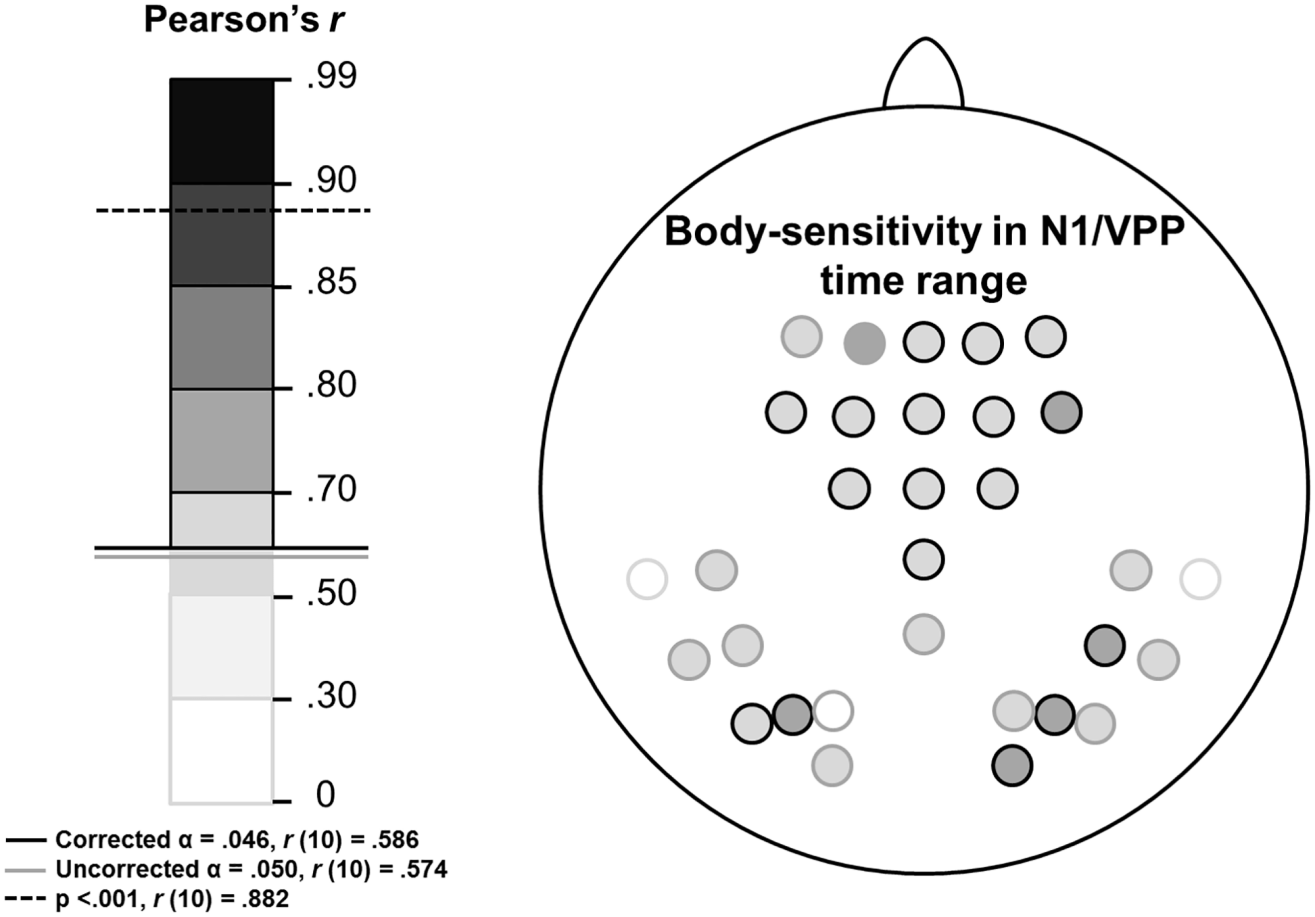
Correlations of body-sensitive N1/VPP effects, between the weeks, at each analysed electrode site are depicted separately above. For each electrode site the weakest correlation (lowest Pearson’s r-value) is depicted out of the six possible combinations of the four weeks. Left panel shows the r-value scale: darker colours indicate stronger relationships. Shades above the solid black line were significant after FDR correction (α = .046). Right panel shows scalp maps of analysed electrodes for each component (posterior electrodes for body-sensitivity over N1 and anterior electrodes for body-sensitivity over VPP). Where all relationships between all weeks were significant the electrode is outlined in black. When at least one non-significant relationship was found between two of the weeks the electrode is outlined in grey.

**Fig 10:**
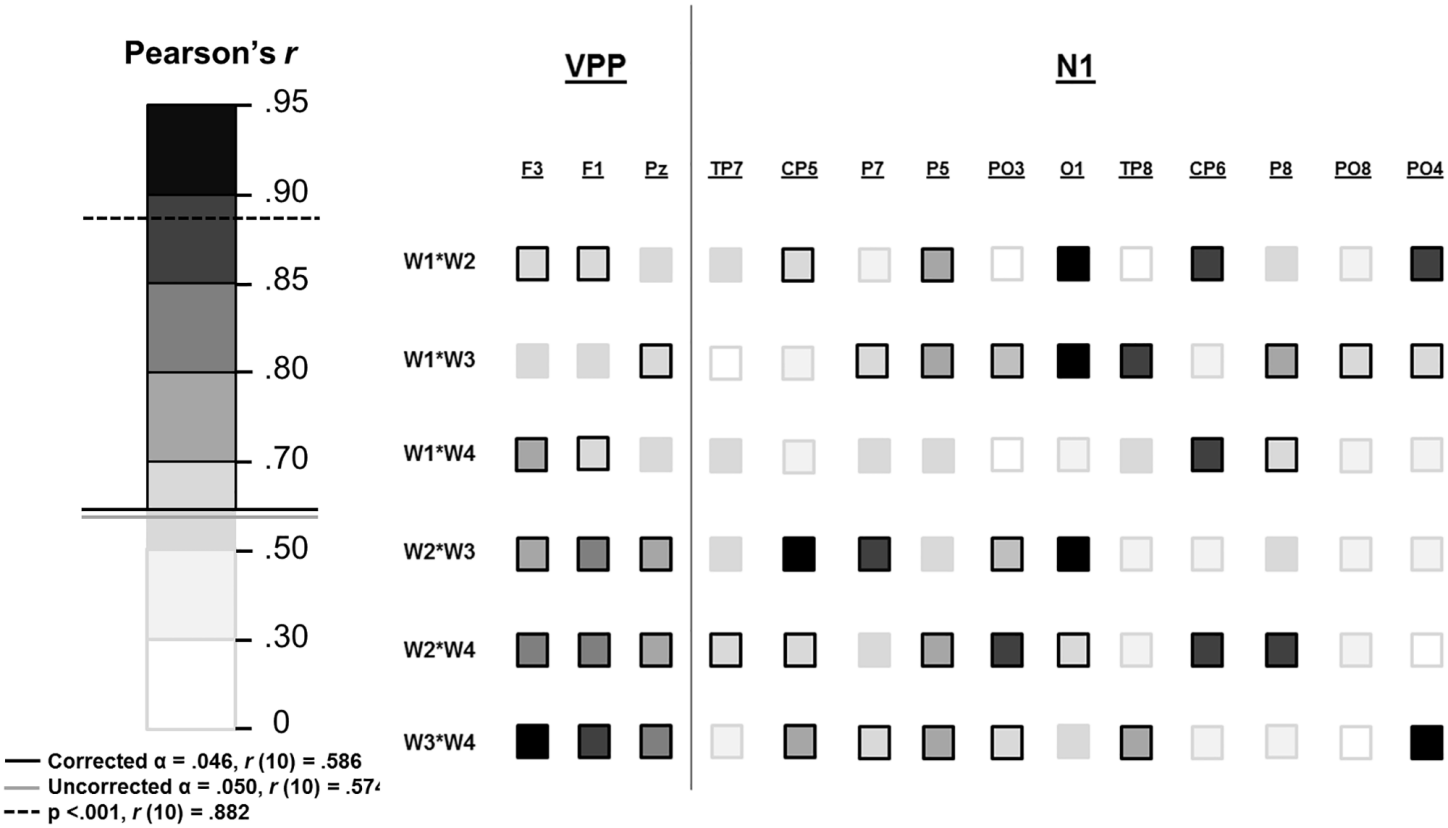
Correlations of body-sensitive effects between weeks at electrode sites that did not yield six significant relationships. Left panel shows the scale pertaining to Pearson’s r values, with darker colours indicative of a stronger relationship. Relationships indicated by a shade above the solid black line were significant after FDR correction (α = .046). Right panel shows separate week-to-week correlations at electrode sites where not all relationships were significant for N/VPP body sensitivity. Non-significant relationships are outlined in grey; significant relationships are outlined in black.

Data from each week were then subjected to separate Pearson’s product-moment correlational analyses at each electrode. Similarly to the previous analysis, six relationships between the weeks, representing each possible pairing of weeks, were therefore obtained at each electrode site separately for N1 and VPP data (see Fig 9). At electrode sites where six significant relationships were not found, the pairings are listed separately in Fig 10. Upon viewing this, it seems that fronto-central body-sensitivity might be a more robust indication of body processing over time, compared to more posterior body-sensitive processing.

#### Between-weeks versus within-weeks comparisons of body-sensitive effects

**Fig 11:**
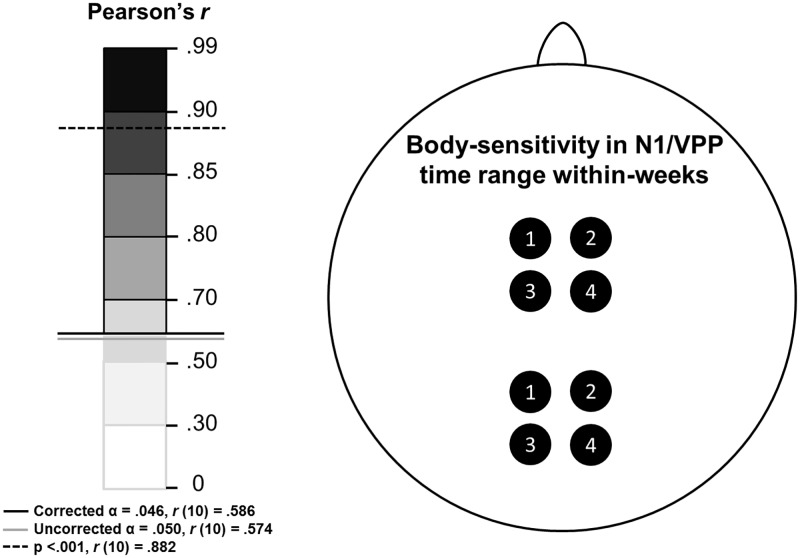
Body-sensitive ERP amplitude correlations within-weeks between the four electrode clusters are depicted above. N1 data for the four weeks are drawn posteriorly, while VPP data are shown anteriorly. For each week, the weakest correlation (lowest Pearson’s r-value) is depicted out of the six possible combinations of the four clusters. Left panel shows the r-value scale: darker colours indicate stronger relationships. Shades above the solid black line were significant after FDR correction (α = .046). Since all relationships between all clusters were significant, the circles are all outlined in black.

**Fig 12:**
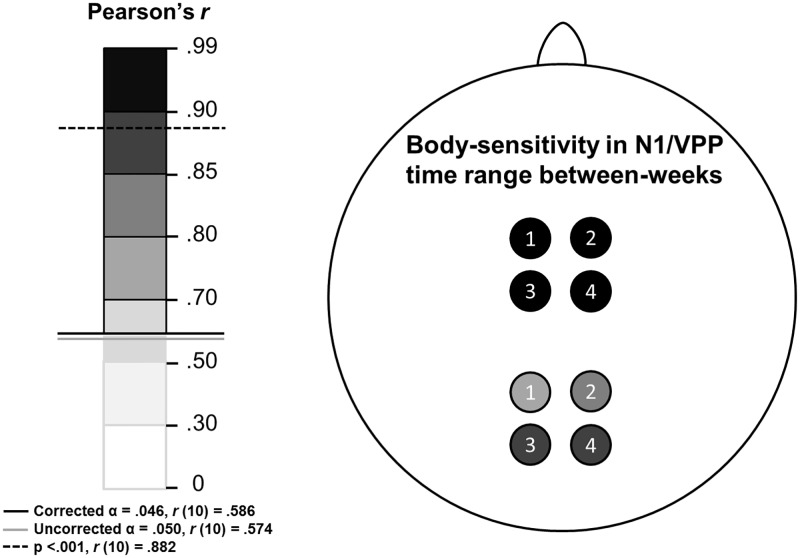
Body-sensitive ERP amplitude correlations between-weeks within the four electrode clusters are depicted above. N1 data for the four clusters are drawn posteriorly, while VPP data are shown anteriorly. For each cluster, the weakest correlation (lowest Pearson’s *r*-value) is depicted out of the six possible combinations of the four weeks. Left panel shows the *r*-value scale: darker colours indicate stronger relationships. Shades above the solid black line were significant after FDR correction (α = .046). Since all relationships between all weeks were significant, the circles are all outlined in black.

Finally, we sought to establish whether our reliable body-sensitive between-week amplitude correlations were comparable in size to within-week correlations between different but overlapping clusters of electrodes. For this analysis, body/house differences were computed at each electrode site and four arbitrary and similar clusters of electrodes were defined: Cluster 1, FC1/2 & CZ & PZ; Cluster 2, F3/4 & C1/2; Cluster 3, F1/2 & FC3/4; and Cluster 4, FZ & FCZ & CPZ. For each of the four clusters and for each of the four weeks the mean amplitude VPP and N1 were calculated for each participant. These data were then correlated in two ways: (A), within each cluster, all six pairs of weeks were correlated, yielding 24 correlations between weeks; and (B), within each week, all six pairs of clusters were correlated, yielding 24 correlations within weeks. Note that, within a given week, all clusters’ amplitude measurements were driven by identical underlying brain activity, and recorded by very similarly located electrodes. Such correlations represent a notional maximum correlation for this experiment. Thus, as expected these within-week, between-cluster correlations were very high (N1: Mean r(12) = .979, SE = .010. VPP: Mean r(12) = .985, SE = .018. See also Fig 11). The analogous N1 and VPP between-week, within-cluster correlations approached our notional maximum values very closely, being 93% and 98% of the within-week *r*-values (N1: Mean *r*(12) = .909, SE = .056. VPP: Mean *r*(12) = .965, SE = .016. See also Fig 12).

## Discussion

The present study aimed to test the reliability, over time, of early visual ERPs and body-sensitive ERP effects. We did so in order to inform the validity of research in the body processing field, especially with respect to whether these body-sensitive components have the potential to be trustworthy neural markers.

Amplitude and latency of P1, N1 and VPP responses to body and house stimuli, recorded over a 4-week period in six men and six women, showed that early visual components, as well as body-sensitive effects, were reliable and stable over time. Specifically, ANOVA has shown that group ERP values are consistent over time, as null effects for all components were reported. Strong correlation co-efficients in the correlational analyses also show that individuals’ ERP values are stable over time. For example, if group averages were stable but individuals’ ERP values were randomly fluctuating, then that would lead to weak correlation coefficients. As this is not the case, we are confident that the observed effects are due to stability, and not random individual changes.

In addition, correlational analyses between the weeks at each electrode site showed that VPP amplitude may be a more robust indication of early visual processing than VPP latency. This is in line with similar findings from previous research on the reliability of other visual, auditory, face-selective and error-related components early-, late- and face-selective visual, auditory and error-related components [26, 40, 42].

Correlational analyses also indicated specific electrode sites that may yield more reliable reflections of body-related N1 and VPP cortical activity, respectively. In particular, body-sensitivity observed over VPP may be more robust than that which is observed more posteriorly, over N1.

### Extending on the understanding of electrophysiological body-sensitive mechanisms

Consistent with previous research [9, 17, 56], an enhancement of electrophysiological activity over occipito-parietal and fronto-central sites was observed in the N1 and VPP time range. This adds to the literature which proposes that bodies, like faces, are processed by specialised cortical areas [14, 57]. With respect to body sensitivity per se, these results support the notion that N1 and VPP responses are generated from the same neuronal sources [16, 20].

We also observed relatively early temporal differences between body and house viewing over lateral occipito-temporal sites (TP7/8, CP5/6, P7/8, and P5/6) such that the P1 peaked earlier in response to bodies as compared to houses. Such rapid category-selective cortical activation has been shown for bodies in at least two other studies [10, 58] but seems to be a relatively rare finding [17]. As we did not observe this effect at all electrode sites, it is possible that the sites over which effects are reported may be at least partly responsible for whether early category-specific activity is uncovered in studies of visual body perception. Moreover, we found body-sensitive P1 latency effects to be most robust over occipito-parietal sites (particularly in the right hemisphere), rather than temporal sites. As the effect in our study was seen specifically over lateral occipito-temporal sites, this too may help to explain why such rapid categorical distinctions are not always observed. Nonetheless, the present study contributes additional evidence for rapid distinctions between bodies and non-body stimuli during the early stages of visual analysis.

Correlational analyses also revealed that the fronto-central body-sensitivity observed in VPP amplitudes might be more robust than the body-sensitive effects observed more posteriorly, over N1, at least with the particular EEG montage we employed in this study [20].

### Early visual components, and early body-sensitive effects, are stable over time

Our findings show that early visual ERP components and the body-sensitive effects within their time ranges are stable over time within the same individuals, in line with what has been reported for auditory, face-selective and error-related ERP components [28, 39, 43]. Moreover, correlations between the weeks at each electrode site for each component showed that amplitude may be more reliable over time than latency. These correlational analyses also revealed that P1 amplitude and latency and VPP latency may be less robust at certain sites. Specifically, our results suggest that anterior (F1/2, F3/4, FC1/2, FC3/4, Fz and Fcz), rather than central or posterior (C1/2, CZ, CPZ, PZ), sites may be the most robust indicators of the cortical activity reflected at VPP. At the same time, occipito-parietal (O1/2, PO3/4, PO5/6, PO7/8, P7/8, P5/6), rather than more temporal (TP7/8, CP5/6), sites (particularly in the right hemisphere) may be the most robust source of cortical processes reflected at the P1-N1 complex. This is a finding of particular significance with regards to the study of visual body perception, as it strongly suggests that there are electrode sites whereby a more reliable indication of cortical activity may be obtained (i.e. electrode sites where all correlations were significant). We therefore suggest that our findings are considered as a potential guide for electrode selection in future studies of visual processing and in particular, visual processing of the human body form using a similar EEG montage.

Previous research has suggested that visual ERPs and body-sensitive ERP effects may provide a potential bio-signature of disorders characterised by body image disturbance [33, 34, 59]. The implications of such claims advocate the use of neural markers to identify ‘at risk’ individuals, or to track the severity of symptoms and the efficacy of treatment over time. However, such possibilities rely on the inherent assumption that the ERPs and their associated effects are stable and reliable biological phenomena that are not subject to random changes within the same individual. For the first time, we provide explicit evidence in support of this assumption. In line with previous findings for other cortical (dys)functions [30, 31], visual P1, N1 and VPP responses, as well as body-sensitive effects, appear to have potential as electrophysiological biomarkers.

## Conclusion

The results of this study show that early visual ERPs and body-sensitive effects are stable over time, with amplitudes relatively more stable than latencies. As a result, we confirm that P1, N1 and VPP responses, as well as their associated body-sensitive effects, are eligible candidates for research into bio-signatures of cognitive (dys)function related to body representation.

The results of this investigation also suggest that future studies of visual perception, and perhaps visual body perception in particular, should carefully consider the electrode sites from which data are analysed, as some sites appear to provide a more robust indication of relevant cortical activity than others.

## Supporting information

S1 File Behavioural data. Reaction time and accuracy data in response to bodies and houses.
(SAV)

S2 File Peak amplitude data. P1, N1 and VPP peak amplitude data recorded over occipito-parietal and fronto-central electrodes in response to other bodies and houses.
(SAV)

S3 File Peak latency data. P1, N1 and VPP peak latency data recorded over occipito-parietal and fronto-central electrodes in response to other bodies and houses.
(SAV)
